# The association between health costs and physical inactivity; analysis from the Physical Activity at Work study in Thailand

**DOI:** 10.3389/fpubh.2023.1037699

**Published:** 2023-03-07

**Authors:** Katika Akksilp, Wanrudee Isaranuwatchai, Yot Teerawattananon, Cynthia Chen

**Affiliations:** ^1^Saw Swee Hock School of Public Health, National University of Singapore and National University Health System, Singapore, Singapore; ^2^Health Intervention and Technology Assessment Programme, Ministry of Public Health, Bangkok, Thailand; ^3^Yong Loo Lin School of Medicine, National University of Singapore and National University Health System, Singapore, Singapore

**Keywords:** physical activity, exercise, economic cost, healthcare, societal cost

## Abstract

**Introduction:**

Physical inactivity increases the risks of several common yet serious non-communicable diseases, costing a tremendous amount of health expenditure globally. This study aimed to estimate the association between health costs and physical inactivity in Thailand.

**Methods:**

Data from the Physical Activity at Work cluster randomized controlled trial participants with valid objective physical activity data were extracted. Health costs were collected using the Health and Welfare Survey and the Work Productivity and Activity Impairment Questionnaire and were categorized into past-month outpatient illness, past-year inpatient illness, and past-week presenteeism and absenteeism. Time spent in moderate-to-vigorous physical activity was used to determine the activity level according to the current guideline (i.e., ≥150 minutes moderate-intensity or ≥75 minutes vigorous-intensity equivalent physical activity per week). The primary analysis evaluated the association between direct cost (treatment and travel costs) and societal cost (direct cost plus absenteeism due to the illness) of past-month outpatient illness and physical inactivity using a two-part model.

**Results:**

In total, 277 participants with a mean age of 38.7 were included. Average direct and societal cost due to past-month outpatient illness were 146 THB (3.99 USD) (SD = 647 THB) and 457 THB (12.5 USD) (SD = 1390 THB), respectively. Compared to active participants, direct and societal cost of past-month outpatient illness were 153 THB (4.18 USD) (95%CI: –54.7 to 360 THB) and 426 THB (11.7 USD) (95%CI: 23.3 to 829 THB) higher in physically inactive individuals, respectively, adjusted for covariates. The additional societal cost of past-month outpatient illness was 145% higher in physically inactive participants compared to active participants. On the other hand, there was no significant association in direct and societal cost of past-year inpatient illness nor past-week indirect costs between physically active and non-active participants.

**Discussion:**

Results were similar to recent findings in different countries. However, the findings should be generalized with caution due to the small sample size and potential bias from reverse causation. Future research is crucial for clarifying the health costs of physical inactivity in Thailand and other countries.

## 1. Introduction

Physical inactivity is a significant risk factor for developing several non-communicable diseases (NCDs), such as metabolic syndromes and cardiovascular diseases (1–4). In 2013, the total cost attributable to physical inactivity was $67.5 billion globally, calculated from five major NCDs: coronary heart disease, stroke, type 2 diabetes, breast cancer, and colon cancer (5). In addition, the lack of physical activity (PA) has recently been found to increase the risks of other NCDs as well as depression and falls (1, 6). These have become common serious issues with increasing prevalence (7–9). Strategies to increase PA have been researched and implemented in several countries at all income levels. However, little success has been observed due to the lack of awareness of the intermediate impact at the individual level, e.g., short-term health and economic consequences from physical inactivity (10).

To direct policymakers' focus on this crucial matter, many studies have recently focused on economic burdens related to physical inactivity (11–14). Various methodological approaches were implemented to estimate health expenditures due to physical inactivity (15). Moreover, it is well established that the economic burden depends significantly on regional cultural differences (16). Therefore, more studies have been conducted to generate better evidence in low- and middle-income countries, where the prevalence of physical inactivity has been increasing rapidly, especially in the urban population (3, 10).

In Thailand, there have been many attempts to increase PA in all age groups, including the National Step Challenge in 2020. This is a nationwide program under the “Thailand Physical Activity Strategy 2018–2030”, a roadmap led by the Ministry of Public Health, Thailand (17). Self-report PA data from Thai studies showed that around three-quarters of the adult population is physically active, as defined by the current guideline (18, 19). On the other hand, a recent report from the Thai National Step Challenge data, translated from built-in smartphone accelerators, depicted a low level of PA with an average of 3,200 steps per day despite being encouraged by the intervention (20).

There has been no explicit estimation of health costs due to physical inactivity in Thailand. Most Thai citizens are covered by either the universal coverage scheme, the government health insurance scheme or the social security scheme (21). This universal health coverage initiative results in fewer patients spending out-of-pocket when using health services. However, the incidence of NCDs has been rising from 15.8 to 17.8% of the population between 2009 and 2019 (22). There is an urgent need for evidence to enhance awareness of policymakers and the general population regarding the intermediate economic impact of physical inactivity on Thai society. This analysis aims to evaluate the association between health costs and physical inactivity using secondary data from a cluster-randomized trial in Thai office workers.

## 2. Methods

### 2.1. Samples

This study used data from the Physical Activity at Work (PAW) cluster-randomized controlled trial. The Ethical Review Committee for Research in Human Subjects, Ministry of Public Health (ECMOPH) (Protocol Number: 004-2563) approved the study in accordance with the Declaration of Helsinki. The trial was registered with the Thai Clinical Trials Registry (TCTR) (TCTR20200604007) (23). More details on the trial can be found in a published trial protocol (24). Participants were recruited between July and September 2020 with the inclusion criteria of: (1) was employed during the study period, (2) aged at least 18 years old, (3) had no physical mobility limitations, (4) worked at least 3 days a week, and (5) owned a smartphone compatible with Fitbit^®^ application. Participants with plans to take leaves of absence for more than 2 weeks and those who were pregnant during the trial were also excluded. In total, 282 office workers in the Ministry of Public Health, Thailand, participated in the study and were allocated by cluster to either the intervention or control group. At the 6-month, 28 participants dropped out from the study resulting in 254 remaining for the follow-up data collection.

### 2.2. Measures

#### 2.2.1. Physical activity

From the PAW data collection, participants were objectively measured PA and sedentary levels by wearing the ActiGraph™ wGT3X-BT tri-axial accelerometer (ActiGraph™, Pensacola, Florida, USA) for 10 days on the waist. The accelerometer was tested as a valid tool for measuring step counts and “a reliable tool for measuring PA in adults under free-living conditions” in recent studies (25, 26). An eligible ActiGraph™ data analysis criteria of wearing the device at least 10 h each day for 3 workdays was implemented to drop invalid data. The research team downloaded the pre-initialized 60 Hz count data from the device using ActiLife 6 software to analyze on RStudio Version 1.4.1103 with the R package “Physical Activity”. Choi's algorithm (27) was used to distinguish wear from non-wear. Freedson's cut-points (28) were used to categorize PA levels as sedentary [<151 counts per minute (CPM)], light-intensity (151–2,689 CPM), moderate-intensity (2,690–6167 CPM), or vigorous-intensity (>6,167 CPM). Finally, time spent in moderate- and vigorous-intensity PA (MVPA) was calculated as: [(daily average of time spent in moderate-intensity PA) + 2 x (daily average of time spent in vigorous-intensity PA)] × 7. Individuals were physically inactive if they had MVPA of <150 min based on the current guideline (29).

#### 2.2.2. Health costs

Economic cost of physical inactivity was estimated from the societal perspective. The Health and Welfare Survey from the National Statistical Office (NSO), a nationwide survey initiated since 1974 for health and welfare reports in Thailand (30, 31), was used as part of the questionnaire to collect health-related expenditures, including out-of-pocket payment of treatment (direct medical cost), travel fees (direct non-medical costs), and absent days (indirect cost) of the past-month outpatient illness and past-year inpatient illness. In spite of the frequent use of the survey data in previous studies (30, 32–34), there has been no validation study of the tool. We use the questionnaire, nevertheless, as it has been used for health and welfare-related research as well as situation analyses in Thailand so that we can compare results effectively with other studies. Regarding indirect costs, an average wage of 20,000 THB (547 USD) per month acquired from the Human Resource office, which was equivalent to 909 THB (24.9 USD) per day or 130 THB (3.56 USD) per hour, was used for the population. We calculated the cost due to absenteeism by multiplying absent days with the daily wage. Eventually, direct and indirect costs were summed up to represent the societal cost. Participants were also asked if their current health insurance scheme covered each payment. If participants were fully covered, the average cost of outpatient care (165 THB; 4.51 USD) and inpatient care (2,944 THB; 80.5 USD), reported by the NSO in 2019 (22), were used to calculate the service fee. Baseline interviews were done between August and September 2020, and follow-up interviews between February and March 2021. All costs are presented in year 2022 dollars.

Moreover, in the sensitivity analysis, we estimated the additional indirect cost due to physical inactivity, calculated as the past-week absenteeism and presenteeism from the Work Productivity and Activity Impairment Questionnaire: General Health V2.0 (WPAI-GH). For absenteeism cost, we asked the absent hours due to health problems (i) and the hours actually worked (ii) during the previous week to calculate the score: (i)/((i) + (ii)). Next, we multiplied the absenteeism score by the average weekly wage (4,546 THB; 126.8 USD). For presenteeism cost, the degree health problems affected work productivity over the last seven days were asked using a rating scale from 0 to 10; 0 for no effect of health problem on work and 10 for being wholly prevented from working. The scale was then used to calculate the cost of past-week presenteeism by the formula; (WPAI-GH presenteeism scale/10) × (number of hours actually worked per week) × (the hourly wage).

#### 2.2.3. Covariates

Questionnaire interviews and physical examinations were done to collect participants' demographics and biomarker data. Covariates included sex, age (continuous, in years), education (highest at bachelor's degree or above bachelor's degree), and obesity (body mass index; BMI ≥ 25).

### 2.3. Statistical analysis

We summarized baseline characteristics by physically active or inactive groups using the mean and standard deviation for continuous variables and the frequencies and percentages for categorical variables. Between-group comparisons of baseline characteristics were done using *t*-test and chi-square test for continuous and categorical variables, respectively.

A two-part model was used to deal with the zero-inflated and the skewness nature of the health cost data (35–37). A probit model first predicted the probability of having health costs. Then, of those with positive health costs, the generalized linear model with a log link and gamma family was used to explore the relationship between health costs and physical inactivity.

In the primary analysis, the model used direct and societal cost of the past-month outpatient illness as the outcome, physically active or inactive as a binary exposure, and adjusted for the covariates. The sensitivity analyses were done for: (i) changing the outcome to direct and societal cost of the past-year inpatient illness; (ii) changing the outcome to the past-week health-related absenteeism and presenteeism from the WPAI-GH questionnaire; and (iii) estimating direct and societal cost using 6-month follow-up data as the outcome, with additional adjustment for baseline costs. We used Stata software version 14.2 for all statistical data analyses, and the significance level was set at 5%.

## 3. Results

The primary analysis involved 277 participants from the PAW study. Participants' mean age was 38.7 years (SD = 10.3), where 81% were women, 23% were obese, and 48% were physically active at baseline. Physically inactive participants appeared to be female (91.7 vs. 69.9%, *p* < 0.001) and older (39.9 vs. 37.3 years, *p* = 0.036). Overall, the average direct and societal cost due to past-month outpatient illness were 146 THB (3.99 USD) (SD = 647 THB) and 457 THB (12.5 USD) (SD = 1,390 THB), respectively (Table 1).

**Table 1:** Baseline characteristics, physical activity levels, and health costs.

	**Total**	**Active^a^**	**Inactive^a^**	***P*-value**
	***N*** = **277**	***N*** = **133**	***N*** = **144**	
Age, year	38.7 (10.3)	37.3 (8.97)	39.9 (11.3)	0.036
Gender, female	225 (81.2%)	93 (69.9%)	132 (91.7%)	< 0.001
Education, above bachelor's degree	98 (35.4%)	48 (36.1%)	50 (34.7%)	0.812
Body mass index (BMI), kg/m^2^	24.4 (5.17)	24.9 (5.40)	24.0 (4.94)	0.128
Obese (BMI ≥ 25 kg/m^2^)	64 (23.1%)	32 (24.1%)	32 (22.2%)	0.717
**Physical activity**				
Moderate physical activity/week, min	24.9 (17.5)	38.1 (16.8)	12.8 (4.82)	< 0.001
Vigorous physical activity/week, min	0.652 (1.64)	1.19 (2.20)	0.152 (0.417)	< 0.001
**Health cost (THB)**				
Direct cost^b^: Past-month outpatient illness	146 (647)	85.6 (464)	201 (777)	0.014
Societal cost^c^: Past-month outpatient illness	457 (1,390)	294 (1,080)	608 (1,620)	0.063
Direct cost^b^: Past-year inpatient illness	1,300 (12,500)	261 (1,840)	2,260 (17,300)	0.158
Societal cost^c^: Past-year inpatient illness	2,110 (18,300)	563 (3,770)	3,530 (25,000)	0.155

Categorical variables are expressed in count (percentage); Continuous variables are expressed in mean (standard deviation).

a Active refers to physically active participants according to the current guideline (≥150 min moderate-intensity or ≥75 min vigorous-intensity equivalent physical activity per week).

b Direct cost included treatment and travel costs.

c Societal cost included treatment, travel costs, and absenteeism due to the illness.

**Figure 1:**
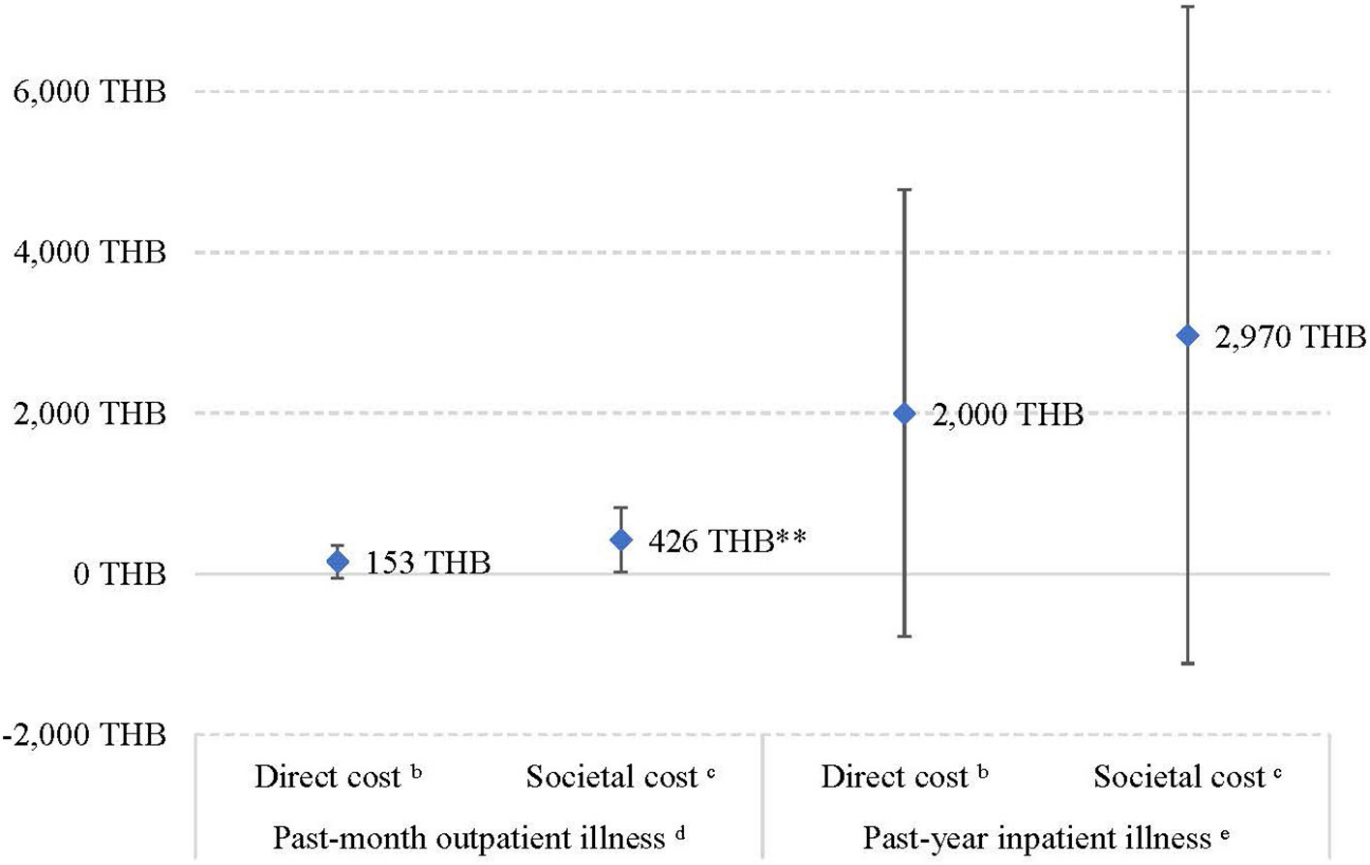
Additional health costs in physically inactive participants compared to active^a^ participants. ^a^Active refers to physically active participants according to the current guideline (≥150 min moderate-intensity or ≥75 min vigorous-intensity equivalent physical activity per week). ^b^Direct cost included treatment and travel costs. ^c^Societal cost included treatment, travel costs, and absenteeism due to the illness. ^d^Part 2 of the two-part model; adjusted for age, sex, obesity, and education. ^e^Part 2 of the two-part model; unadjusted because only 14 participants reported having past-year inpatient illnesses. **p < 0.05.

Compared to physically active participants, additional direct and societal cost of past-month outpatient illness due to physical inactivity were 153 THB (4.18 USD) (95%CI: −54.7 to 360 THB) and 426 THB (11.7 USD) (95%CI: 23.3–829 THB), respectively (Figure 1; Tables 2, 3). The additional societal cost of past-month outpatient illness in inactive participants could be calculated as a 145% increase compared to physically active participants. Nevertheless, while physically inactive individuals had 2,000 THB and 2,970 THB higher direct and societal cost, respectively, in the past-year inpatient illness, this finding was not statistically significant (Figure 1).

**Table 2:** Adjusted analysis details of the difference in direct cost^a^ of past-month outpatient illness between physically active and inactive participants (THB).

	**Unadjusted model**	**Adjusted for + demographics**	**Adjusted for + education**
	**Beta (SE)**	**Beta (SE)**	**Beta (SE)**
Inactive^b^	116 (79.1)	138 (98.2)	153 (106)
Female		65.0 (89.9)	53.0 (101)
Age, year		−1.85 (5.05)	−2.29 (5.19)
Obese (BMI ≥ 25 kg/m^2^)		271 (186)	265 (188)
Highest education: above bachelor's degree			43.7 (103)
Observations	277	277	277

a Direct cost included treatment and travel costs.

b Inactive refers to physically inactive participants according to the current guideline (<150 min moderate-intensity or <75 min vigorous-intensity equivalent physical activity per week).

**Table 3:** Adjusted analysis details of the difference in societal cost^a^ of past-month outpatient illness between physically active and inactive participants (THB).

	**Unadjusted model**	**Adjusted for + demographics**	**Adjusted for + education**
	**Beta (SE)**	**Beta (SE)**	**Beta (SE)**
Inactive^b^	314^*^ (169)	425^**^ (198)	426^**^ (206)
Female		150 (185)	151 (193)
Age, year		−24.2^**^ (10.3)	−23.7^**^ (10.5)
Obese (BMI ≥ 25 kg/m^2^)		664^**^ (328)	659^**^ (328)
Highest education: above bachelor's degree			−61.1 (171)
Observations	277	277	277

a Societal cost included treatment, travel costs, and absenteeism due to the illness.

b Inactive refers to physically inactive participants according to the current guideline (<150 min moderate-intensity or <75 min vigorous-intensity equivalent physical activity per week).

* p < 0.10.

** p < 0.05.

Regarding indirect costs due to the past-week health-related presenteeism and absenteeism from the WPAI-GH questionnaire, the average cost was 982 THB (26.9 USD) (SD = 1,290 THB). Around 85.9% of this was from the presenteeism cost. The additional indirect cost due to physical inactivity was 271 THB (7.41 USD) (95%CI: −51.1 to 593 THB), adjusted for covariates (Supplementary Tables S1, S2).

In the adjusted analyses for both the societal cost of the past-month outpatient illness and the additional cost due to past-week health-related presenteeism and absenteeism due to physical inactivity, having obesity (BMI ≥ 25 kg/m^2^) was significantly associated with additional health costs (Table 3; Supplementary Table S3).

The sensitivity analysis showed that 89 participants were physically inactive at both baseline and follow-up time points, 26 participants were physically active at baseline but became inactive at follow-up, 36 participants were physically inactive at baseline but became active at follow-up, and 94 participants were physically active at both time points. We found no evidence in different health costs of past-month outpatient illness at follow-up among different PA change categories (Supplementary Table S4; Supplementary Figure S1).

## 4. Discussion

This study was the first study in Thailand to research the associations between health costs and physical inactivity using the PAW cluster-randomized controlled trial data. The primary analysis showed evidence of a 145% increase in societal cost among those with physical inactivity compared with active participants. The result aligned with previous research in other countries (38, 39) that being physically active was associated with a lower health cost. Using mean annual health expenditure as the outcome variable, Carlson et al. found 30% higher health expenditure in physically inactive US adults compared to active adults, and Brown et al. found 26% higher health expenditure in sedentary than in moderately active Australian women (40, 41). However, comparing findings between studies is challenging because of the different methods of assessment and estimation of the association between cost outcomes and physical inactivity, including the general context of study settings (15, 40).

Most studies reported health expenditures that included the payer's perspective and neglected direct non-medical and indirect costs (15, 42–44). While it is true that these studies extracted extensive variables from linked databases, the challenge remained to extrapolate societal cost. In our study, keeping in mind the small sample size, we analyzed both the direct costs and the societal cost of different components of health costs, inspiring further evidence generation for informing policy and practice in the future.

Indirect cost is a crucial component in health economic studies and frequently constitutes a significant amount of health costs in various diseases (45–47). Different methods have been used to evaluate indirect costs, including different data collection tools and parameters such as disease categories and gross domestic product (GDP) per capita, resulting in high variance and obstacles to comparing studies (48, 49). We used the validated WPAI-GH questionnaire (50) and estimated costs due to the past-week of health-related absenteeism and presenteeism aligned with the commonly used technique, i.e., the human capital approach. Compared to the cost of the past-month outpatient illness, the past-week indirect cost was higher, with a considerable proportion from presenteeism, which was found in other studies (46, 49, 51–53). However, estimating indirect cost have always been a controversial issue and might result in overestimation. Hence, standardization of the methodology in future evaluation and reporting is needed (54–57).

Reverse causality is a potential issue in this study because the primary analyses used cross-sectional data where both PA and health costs data were collected at the same time (baseline), unlike some studies, which incorporated a lag of cost data collection succeeding the PA data extraction (40). Nevertheless, the PAW study inclusion criteria addressed the issue by recruiting participants without physical mobility limitations (24). In addition, we analyzed associations between health costs of the past-month outpatient illness at the follow-up time point and the changes in PA level from baseline to follow-up. However, this sensitivity analysis found no significant association (Supplementary Table S4).

A strength of our analysis other than the previously mentioned corporation of different cost components was that, while other studies use self-report or prevalence estimates of physical inactivity (15), we used objectively measured PA levels from a standardized tool (ActiGraph^TM^) with validation processes. However, this study has some limitations; firstly, only 277 observations were included in the primary analysis. In order to detect the reported between-group difference, with a standard deviation of 645, a minimum sample size of 495 participants per group is required. Compared to most studies evaluating associations between health costs and physical inactivity, our sample size was relatively small. Moreover, PAW participants were office workers from the Ministry of Public Health, Thailand, who were presumably more health conscious, limiting generalizability. Secondly, we used self-report data from the PAW study to estimate health costs, including out-of-pocket payment for services, travel fees, and days absent due to the illness, which may result in more information bias compared to most studies using health expenditure data from national databases (15). Thirdly, due to the way questions were asked in the Health and Welfare Survey, it was not possible to combine different cost components as they could refer to different illnesses (e.g., the societal cost of past-month outpatient illness and past-year inpatient illness). This hindered reporting annual health costs, thus, preventing comparison among studies from different contexts. More sophisticated cost data will be required for future studies to advance health economic research.

## 5. Conclusions

There was evidence of a positive association between the societal cost of the past-month outpatient illness and physical inactivity. The change in PA level might not be large enough to detect the change in health costs within the next follow-up period. These additional analyses of the PAW trial provided important evidence for public health communication and future policy advocacy. Future PA studies, including experimental as well as observational designs, should incorporate the economic cost of health data collection to comprehensively evaluate the associations between the change in health costs and the change in PA levels.

## Data availability statement

During the study, only de-identified data were used, and the data were only accessible to the research team. The research team will have exclusive rights to the de-identified data for 24 months after the trial is completed. After that, the data and full protocol will be publicly accessible on the HITAP website.

## Ethics statement

The studies involving human participants were reviewed and approved by the Ethical Review Committee for Research in Human Subjects, Ministry of Public Health, Thailand. The patients/participants provided their written informed consent to participate in this study.

## Author contributions

KA was Principal Investigator (PI) of the trial. KA, YT, WI, and CC drafted the manuscript together. WI and CC provided statistical expertise. YT, WI, and CC provided expertise on economic analyses. All authors contributed to the study design, reviewed the manuscript draft, have read, and approved the final version.
