# Supplementary material for: The association between health costs and physical inactivity; analysis from the Physical Activity at Work study in Thailand
Source: Front Public Health. 2023 Mar 7;11:1037699. doi: 10.3389/fpubh.2023.1037699 (PMC10027789; doi:10.3389/fpubh.2023.1037699)
Supplement: Supplementary file 1 [file Table_1.DOCX]

Supplementary Material

| **Table S1.** Cost of past-week absenteeism and presenteeism (THB) | | | | |
| --- | --- | --- | --- | --- |
|  | Total | Active ^a^ | Inactive ^a^ | P-value |
|  | N=277 | N=133 | N=144 |  |
| Cost of past-week absenteeism | 139 (521) | 95.3 (331) | 180 (648) | 0.160 |
| Cost of past-week presenteeism | 842 (1150) | 746 (1100) | 931 (1190) | 0.17 |
| Cost of past-week absenteeism and presenteeism | 982 (1290) | 841 (1180) | 1110 (1380) | 0.080 |
| The continuous variables are expressed in mean (standard deviation)  ^a^ Active refers to physically active participants according to the current guideline (≥150 minutes moderate-intensity or ≥75 minutes vigorous-intensity equivalent physical activity per week) | | | | |

| **Table S2.** The difference in cost of past-week absenteeism and presenteeism between physically active and inactive participants (THB) | | | | | | |  | |
| --- | --- | --- | --- | --- | --- | --- | --- | --- |
|  |  | | Mean difference  (Inactive – Active) ^a^ | | Adjusted Mean difference  (Inactive – Active) ^a^ | | |  |
| Outcome |  |  | β (95% CI) ^b^ | P-value | β (95% CI) ^c^ | P-value |  |  |
| Cost of past-week absenteeism |  |  | 84.9  (-33.6 – 203) | 0.160 | 67.8  (-61.1 – 197) | 0.303 |  |  |
| Cost of past-week presenteeism |  |  | 186  (-84.0 – 456) | 0.177 | 204  (-81.1 – 490) | 0.161 |  |  |
| Cost of past-week absenteeism and presenteeism |  |  | 271  (-31.0 – 573) | 0.079 | 271  (-51.1 – 593) | 0.099 |  |  |
| ^a^ Active refers to physically active participants according to the current guideline (≥150 minutes moderate-intensity or ≥75 minutes vigorous-intensity equivalent physical activity per week)  ^b^ Two-part models (unadjusted)  ^c^ Two-part models; adjusted for age, sex, obesity, and education | | | | | | |  | |

| **Table S3.** Adjusted analysis details of the difference in cost of past-week absenteeism and presenteeism between physically active and inactive participants (THB) | | | |
| --- | --- | --- | --- |
|  | Unadjusted model | Adjusted for +Demographics | Adjusted for  +Education |
|  | beta (SE) | beta (SE) | beta (SE) |
| Active ^a^ | 0.0 | 0.0 | 0.0 |
|  | (.) | (.) | (.) |
| Inactive | 271^*^ | 263 | 271^*^ |
|  | (154) | (162) | (164) |
| Female |  | 0.0 | 0.0 |
|  |  | (.) | (.) |
| Male |  | -131 | -119 |
|  |  | (212) | (213) |
| Age, year |  | -11.2 | -13.4^*^ |
|  |  | (7.73) | (7.90) |
| Normal BMI |  | 0.0 | 0.0 |
|  |  | (.) | (.) |
| Obese (BMI ≥ 25 kg/m^2^) |  | 486^**^ | 506^**^ |
|  |  | (219) | (219) |
| Highest education: bachelor's degree |  |  | 0.00 |
|  |  |  | (.) |
| Highest education: above bachelor’s degree |  |  | 263  (172) |
| Observations | 277 | 277 | 277 |
| ^a^ Active refers to physically active participants according to the current guideline (≥150 minutes moderate-intensity or ≥75 minutes vigorous-intensity equivalent physical activity per week)  ^*^ *p* < 0.10, ^**^ *p* < 0.05 | | | |

| **Table S4.** Different health costs of the past-month outpatient illness at 6-month follow-up among physical activity change categories | | | | | | |  |
| --- | --- | --- | --- | --- | --- | --- | --- |
|  | Baseline | Follow-up | Mean difference | | Adjusted Mean difference | | |
| Outcome | Mean (SD) | Mean (SD) | β (95% CI) ^d^ | P-value | β (95% CI) ^e^ | P-value |  |
| Direct cost (THB) ^a^  - Inactive > Inactive ^c^  (n = 89) | 152  (493) | 138  (298) | Reference |  | Reference |  |  |
| - Active > Inactive  (n = 26) | 40.8  (76.3) | 267  (985) | 130  (-162 – 421) | 0.384 | 208.4  (-129.8 – 546.6) | 0.227 |  |
| - Inactive > Active  (n = 36) | 183  (558) | 126  (251) | -12.0  (-148 – 124) | 0.863 | 22.4  (-115.8 – 160.7) | 0.750 |  |
| - Active > Active  (n = 94) | 108  (549) | 139  (393) | 1.20  (-103 – 105) | 0.982 | -17.4  (-101.9 – 67.0) | 0.686 |  |
| Societal cost (THB) ^b^  - Inactive > Inactive  (n = 89) | 484  (1230) | 434  (1060) | Reference |  | Reference |  |  |
| - Active > Inactive  (n = 26) | 338  (456) | 372  (1020) | -61.7  (-506 – 383) | 0.785 | -176  (-599 – 247) | 0.414 |  |
| - Inactive > Active  (n = 36) | 713  (2190) | 403  (965) | -30.5  (-431 – 370) | 0.881 | -129  (-527 – 268) | 0.524 |  |
| - Active > Active  (n = 94) | 263  (1220) | 387  (953) | -46.7  (-340 – 246) | 0.755 | -148  (-465 – 168) | 0.359 |  |
| ^a^ direct cost included treatment and travel costs  ^b^ Societal cost included treatment, travel costs, and absenteeism due to the illness  ^c^ Active refers to physically active participants according to the current guideline (≥150 minutes moderate-intensity or ≥75 minutes vigorous-intensity equivalent physical activity per week); Inactive 🡪 Inactive refers to having inadequate physical activity level at baseline and follow-up timepoints  ^d^ Part 2 of the two-part model with healthcare cost as the outcome among those with positive healthcare costs comparing active and inactive participants (unadjusted)  ^e^ Part 2 of the two-part model; adjusted for baseline health cost of the past-month outpatient illness, age, sex, obesity, and education | | | | | | |  |
