# Supplementary material for: The association between health costs and physical inactivity; analysis from the Physical Activity at Work study in Thailand
Source: Front Public Health. 2023 Mar 7;11:1037699. doi: 10.3389/fpubh.2023.1037699 (PMC10027789; doi:10.3389/fpubh.2023.1037699)
Supplement: Supplementary file 2 [file Image_1.pdf]

Figure S1. Mean societal cost[a] of past-month outpatient illness among different physical activity categories at baseline and 6-month follow-up

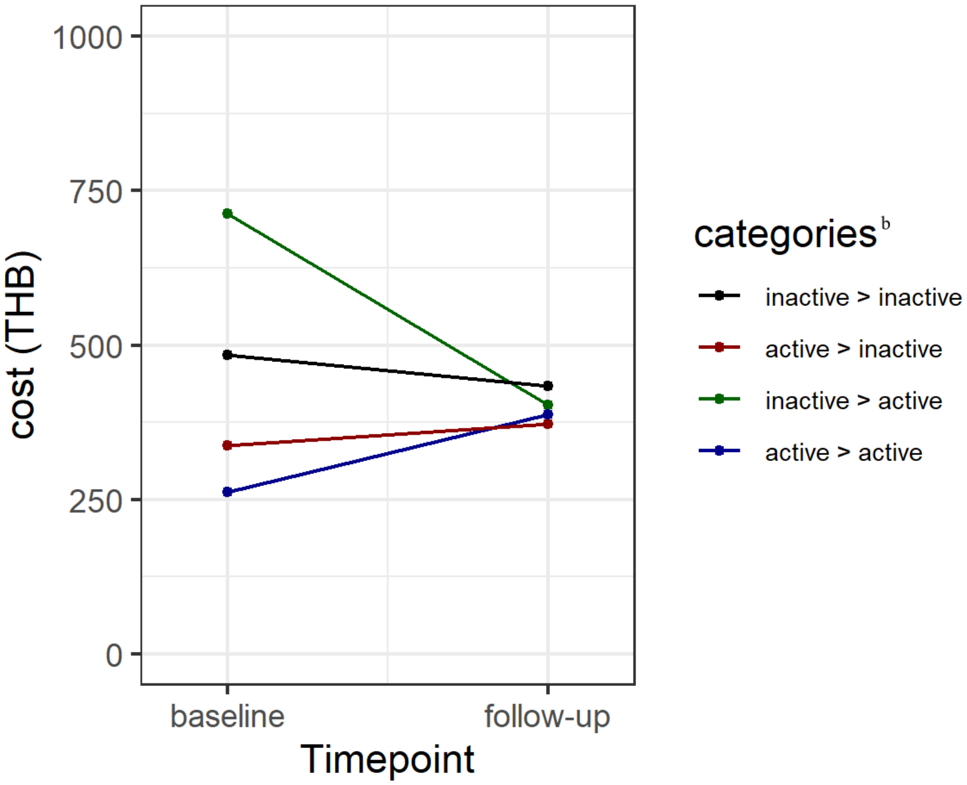

[a] societal cost included treatment, travel costs, and absenteeism due to the illness
[b] categories: "Active" refers to physically active participants according to the current guideline (≥150 minutes moderate-intensity or ≥75 minutes vigorous-intensity equivalent physical activity per week); "active > inactive" refers to participants who were physically active at baseline but inactive at follow-up
